# Designing cost efficient buffer zone programs: An application of the FyrisSKZ tool in a Swedish catchment

**DOI:** 10.1007/s13280-015-0627-y

**Published:** 2015-02-15

**Authors:** Dennis Collentine, Holger Johnsson, Peter Larsson, Hampus Markensten, Kristian Persson

**Affiliations:** 1 Department of Soil and Environment, Swedish University of Agricultural Sciences (SLU), Box 7014, 75007 Uppsala, Sweden; 2 SÖRAB, Box 63, 18621 Vallentuna, Sweden; 3 Department of Aquatic Science and Assessment, Swedish University of Agricultural Sciences (SLU), Box 7050, 75007 Uppsala, Sweden

**Keywords:** Buffer strips, Transaction costs, Baltic Sea, Payment for Environmental Services (PES), FyrisCOST

## Abstract

Riparian buffer zones are the only measure which has been used extensively in Sweden to reduce phosphorus losses from agricultural land. This paper describes how the FyrisSKZ web tool can be used to evaluate allocation scenarios using data from the Svärta River, an agricultural catchment located in central Sweden. Three scenarios are evaluated: a baseline, a uniform 6-m-wide buffer zone in each sub-catchment, and an allocation of areas of buffer zones to sub-catchments based on the average cost of reduction. The total P reduction increases by 30 % in the second scenario compared to the baseline scenario, and the average reduction per hectare increases by 90 % while total costs of the program fall by 32 %. In the third scenario, the average cost per unit of reduction (€163 kg P^−1^) is the lowest of the three scenarios (58 % lower than the baseline) and has the lowest total program costs.

## Introduction

Mobilization and transport of nutrients from terrestrial systems to rivers, lakes, and marine environments cause deteriorating water quality and eutrophication. In Europe, inland water quality is regulated under the European Water Framework Directive (WFD), and in Sweden, each of the five Swedish Water Districts is responsible for ensuring good water status including non-eutrophic status. *No eutrophication* is one of the 16 environmental quality objectives adopted by the Swedish Parliament. In a recent study by the Swedish Environmental Protection Agency, it was identified as an objective that will not be achieved by the target year 2020 under the current set of policies (SNV 2012). Eutrophication is a problem in not only many inland waters in Sweden but also the Baltic Sea. Eutrophication is a problem for the three districts draining to the Baltic Sea. The Northern Baltic Sea District estimates that around 48 % of the water in the district is eutrophic (NBWD (Northern Baltic Sea District) 2008). The Swedish government is committed as a signatory to the Baltic Sea Action Plan (BSAP) to reduce nutrient loads to the Baltic Sea to achieve good environmental status by 2021 (SNV 2008). Measures to reduce nutrient loads need to be implemented to meet the demands of the Swedish environmental quality objective “*No eutrophication*,” the WFD and the BSAP. Unfortunately, controlling nutrient losses has been more difficult than anticipated due to the diffuse nature of the loads; of the total anthropogenic phosphorus loads from Sweden, 40 % originate from farmland (Brandt et al. 2009). Measures to reduce phosphorus loads from the agricultural sector and an increased focus on cost efficiency will be needed to meet reduction targets (SNV 2006).

Riparian buffer zones are the only measure which has been used extensively in Sweden to reduce phosphorus losses from agricultural land. Buffer zones primarily lower phosphorus losses through reducing erosion of particulate P from fields. The effectiveness of a riparian buffer zone depends on the parameters which have an effect on surface runoff (among others topography, soil type, climate, and width of the buffer) and the phosphorus load to the zone. Implementation of riparian buffer zones in Sweden has been supported by payments to landowners from the EU Rural Development Program (RDP). These payments have been a uniform reimbursement per hectare for buffer zones from 6 to 20 m wide in eligible areas for a 5-year commitment. The reimbursement is compensation for the average loss of income for developing the zone and taking the land out of agricultural production, the opportunity cost of the land. This opportunity cost is related to the productivity of the land and varies to a large extent based on agronomic factors. The level of payments has led to uneven and low participation in the program (SLU 2010).

The purpose of this study is to demonstrate how a low transaction cost tool can be used to evaluate and design cost efficient programs for implementation of buffer zones. The first section below describes the design of agri-environmental schemes with a focus on targeting to improve cost efficiency. This section includes a discussion of transaction costs and their impact on policy design and how models by reducing these types of costs can improve the total cost efficiency of programs. The following section describes a web tool developed for evaluating the cost efficiency of buffer zones on reducing phosphorus losses from agricultural land in Sweden, FyrisSKZ. The next section demonstrates how the FyrisSKZ web tool can be used for program evaluation by using the model to compare the cost efficiency of three program scenarios in the Svärta River catchment in Southern Sweden. The paper ends with discussion and conclusions based on the results from the application.

### Design of agri-environmental schemes

There are agri-environmental schemes in place in many countries in the Baltic Sea Region that are designed to reduce the excess nutrient loads reaching the Baltic Sea from agricultural land. In Sweden, a large number of these schemes have been financed through the EU RDP. However, when the effectiveness of RDP program was recently evaluated by the European Court of Auditors, there were many criticisms of ongoing schemes and recommendations were made to the EU Commission with respect to oversight of these programs. In the report published by the Court of Auditors, “Is agri-environment support well designed and managed?” recommendations by the authors to the Commission for the next RDP planning period include that

- agri-environmental expenditures should be more precisely targeted;
- there should be a higher rate of EU contribution for sub-measures with a higher environmental potential;
- there should be a clear distinction between simple and more demanding agri-environment sub-measures;
- and that the Member States should be more proactive in managing agri-environment payments.

(European Court of Auditors 2011).

Researchers that have worked with policy evaluation and development recognize that mitigation measures that can be applied based on site-specific characteristics are cost effective. However, most current mitigation measures are generally regional programs based on broad classes of eligibility with uniform payments for the environmental services provided. To allow for more management flexibility, programs are needed which are able to effectively target the right place with the right measure. However, there is a trade-off between the costs for the site-specific information needed and the increase in effects which come for targeting sites where measures have a greater impact.

Transaction costs involve the costs of running the economic system; the costs of information, contracting, and control. The range of discussion with respect to transaction costs covers a wide scope of economic behavior. Coase (1960) suggested that transaction costs can explain how firms are organized, while economic historian Douglas North (1990) uses the concept to trace the evolution and development of the American economy. A great deal of the literature has focused on the costs associated with the transfer of ownership of a private good and as a corollary to this, property rights. Stavins (1995) suggests that transaction costs are always present in markets “and can arise from the transfer of any property right because parties to exchange must find one another, communicate and exchange information” (p. 134).

With respect to the production of environmental goods and services in agriculture, Rørstad et al. (2007, p. 1) pointed out “the cost of managing a policy may be as important for efficiency as the cost of producing the goods and services.” Some empirical studies estimating the transaction costs of environmental policy show a wide range; as low as 1 % of the production cost of the environmental service provided to as high as 110 % of the production cost (Falconer and Whitby 1999; Falconer and Saunders 2002). But access to information on the transaction costs of environmental policy is limited (Krutilla and Krause 2010). Rørstad et al. (2007) evaluated the transaction costs and impact on cost efficiency of 12 different agricultural policy measures in Norway differentiated by degree across three criteria: the point of application, the degree of asset specificity, and transaction frequency. The first of these criteria is based on the specific characteristic which serves as the metric for a policy, for example, an area subsidy for participating in a particular practice. The second includes those specific characteristics which differentiate one asset from another, for example, the soil type or slope at a specific site. The transaction frequency indicates how often an identical transaction takes place, if a payment for environmental services (PES) policy is able to treat potential sellers identically that this would indicate a high frequency of transactions. While the 12 policies evaluated by Rørstad et al. (2007) spanned a wide range (including taxes, area payments, price supports, and site-specific PES), the main conclusions were that using the three criteria provided robust results for explaining the degree of variance in transaction costs between policies and that “there is a trade-off between transaction costs and precision of the scheme” (p. 10).

When more information is needed, increasing transaction costs can be offset through the use of simple models that summarize available information. Decision Support Systems (DSS) are designed to reduce transaction costs; this includes the use of tools which can provide access to series of harmonized databases. The models described in the following section represent this type of tool and serve to provide information about the cost efficiency of buffer zones targeted at the reduction of P losses from the agricultural landscape. The low transaction costs associated with this tool allow users to evaluate allocation scenarios at a high level of resolution and achieve low cost targeting.

## Materials and methods

The FyrisSKZ model is a web tool which summarizes the cost efficiency of buffer zones along lakes, watercourses, and ditches for all 12 864 sub-catchment areas of Sweden. FyrisSKZ is a public domain web application (at http://fyrisskz.slu.se) which allows users to choose one or several sub-catchments from a GIS interface and view the estimated reduction of a buffer zone on phosphorus losses to the watercourse from surrounding fields, the opportunity and maintenance costs for buffer zones, and the potential area of buffer zones in the chosen sub-catchment. These estimates are presented for five individual buffer zone widths to allow the user to study the influence of the selected width on the reduction effect, costs, and potential area. The web tool was developed under an assignment from the Swedish Water Authorities to provide user friendly access on the cost efficiency of buffer zones based on data from the FyrisCOST DSS. Both the FyrisSKZ and FyrisCOST models were developed by the SLU WaterHUB group at the Swedish University of Agricultural Sciences (http://www.slu.se/en/collaborative-centres-and-projects/slu-water-hub/).

**Fig. 1:**
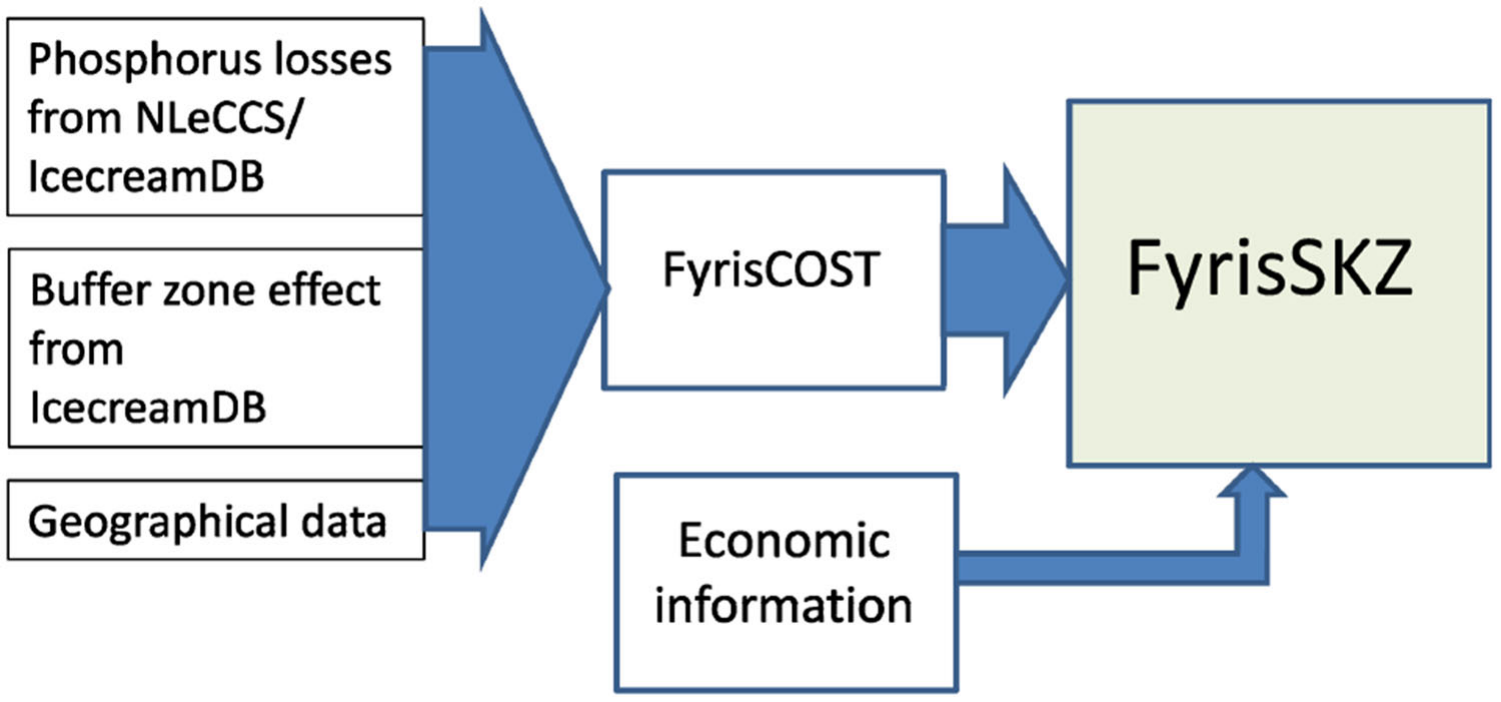
FyrisSKZ model data flow diagram

The results presented in FyrisSKZ are derived from two sources: input entered directly into FyrisSKZ and data imported into the tool from FyrisCOST DSS. As illustrated in Fig. 1, bio-physical information on phosphorus losses from agricultural land use, the reduction effect of buffer zones, and geographical information enter into the FyrisSKZ tool through FyrisCOST. However, economic information on the costs associated with establishing a buffer zone is directly entered into FyrisSKZ.

Data used in the FyrisCOST DSS are derived from an array of field to catchment scale models. Nutrient losses are derived from simulations by the Nutrient Leaching Coefficient Calculation System (NLeCCS) which includes the ICECREAMDB (Larsson et al. 2007) model for estimating P leaching. NLeCCS calculates P losses from agricultural land based on region, soil type, and crop distribution as reported to the national agency for official statistics (Statistics Sweden). Results from NLeCCS are also used in Sweden for the required reporting of nutrient losses to the Baltic Marine Environment Protection Commission (HELCOM) as a part of the Pollution Load Compilations (PLC) performed every 6 years by all countries around the Baltic Sea to protect the marine environment through intergovernmental cooperation (Brandt et al. 2009). The NLeCCS data used for calculating PLC include geographical delineation of catchment and sub-catchment areas, average runoff, area of agricultural land use (block data), area of pasture land use, soil class (FAO class), data on slope (three classes), and soil P (three classes). However, additional geographical data are needed to calculate the spatial impact of buffer zones as a mitigation measure.

To calculate the P reduction effect of a buffer zone in a particular site in the landscape, there needs to be information on the impact area, the length of the impact area along receiving water, and the impact area of existing buffer zones. A land use national database which shows all agricultural fields within 30 m of a lake, watercourse, or ditch is combined with the other inputs in the FyrisCOST DSS to estimate the maximum potential buffer strip and impact area in each sub-catchment. The size of the area within agricultural blocks which are close to water courses and therefore of interest for a buffer zone to reduce P losses is calculated in FyrisCOST using a crop distribution module in the model. Agricultural blocks with managed pasture or extensive pasture, undefined crops, and minor crops are excluded from the potential impact area of buffer zones. Pastures are excluded because of the very limited impact these have on P losses. The latter two land use categories are excluded as there is not enough information for these crops to estimate reliable P loss coefficients in the NLeCCS model.

Identification of possible locations for buffer zones in sub-catchments was carried out using GIS tools (PostgreSOL/PostGIS, ArcGIS, and QGIS) on the agricultural block data. All water courses were assigned a 15-m width and converted to lines in the landscape using the “st_boundary” function in PostgreSOL/PostGIS. Water courses (including ditches) which had a length of less than 30 m along an agricultural block were excluded from the potential area in the sub-catchment. The potential impact area was assumed to be the area in those non-excluded agricultural blocks that was within 50 m of a watercourse, and a 60 m buffer from the watercourse was used to delineate this area.

**Fig. 2:**
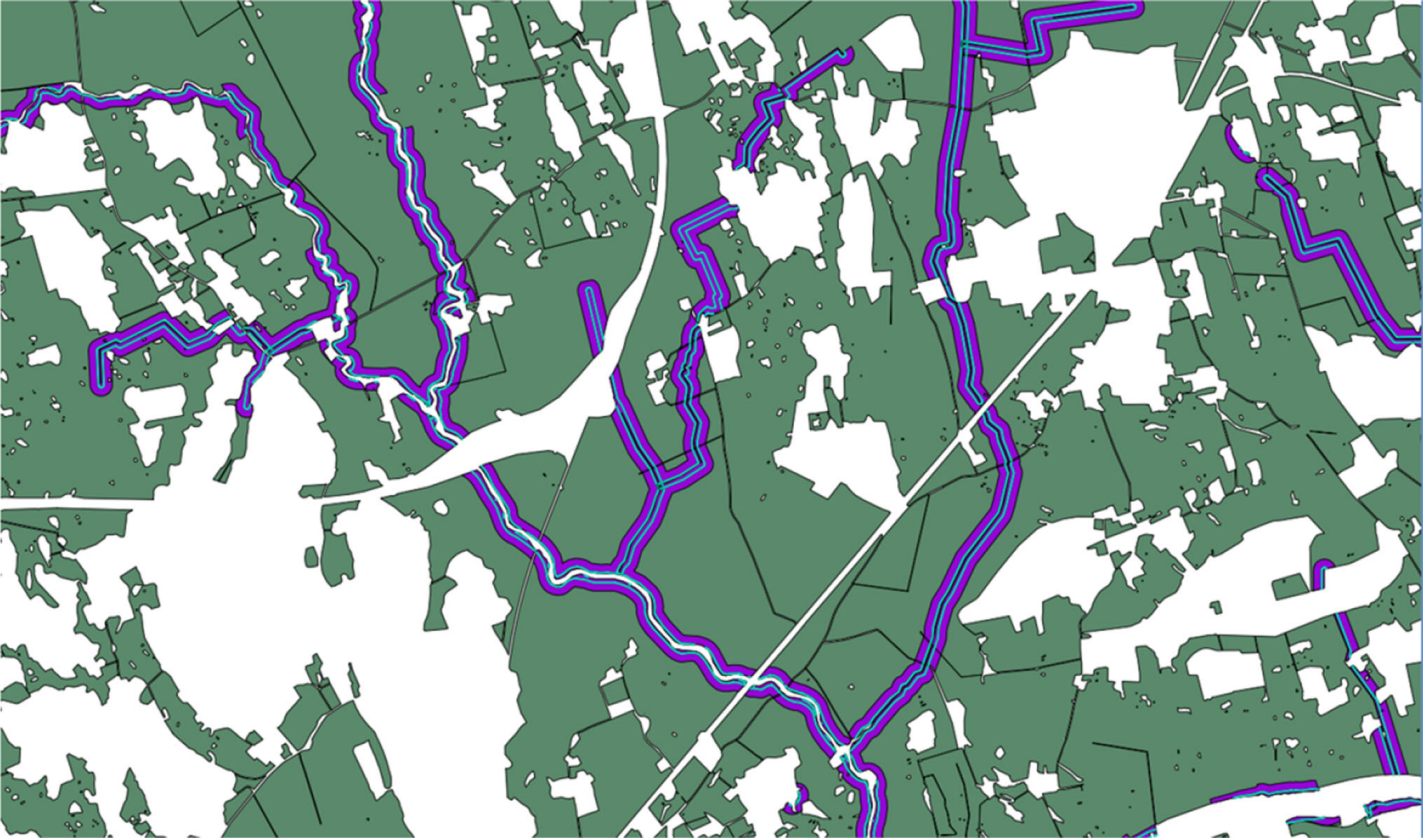
A section of a sub-catchment used for identifying the area of potential buffer zones, *green areas* are agricultural land use, *white areas* non-agricultural land use, *dark blue lines* indicate watercourses, *light-blue lines* along the water courses are areas for potential placement of buffer zones, *purple areas* are the impact areas used to calculate the potential P reduction of a buffer zone

Figure 2 illustrates a section of the mapping of the potential area for buffer zones in one area of a sub-catchment. The green areas in Fig. 2 are agricultural blocks, while the white areas are non-agricultural land use (primarily urban and forest areas). Water courses are marked as a dark blue line which includes the 15 m buffer described above. The purple areas in the figure are impact areas for calculating the reduction of a potential buffer zone along the water courses. The light-blue line indicates the area for potentially placing buffer zones. As can be seen in the several areas in Fig. 2 where there is no agricultural land (land that has been excluded due to its land use), there is no potential for a buffer zone. The white areas within the water course in the center and upper left quadrant in Fig. 2 are an assignment of land use and do not have an effect on the potential area of buffer zones.

For statistical purposes, Sweden is divided into eight production regions where agricultural land within each of the regions has similar production capabilities. The FyrisCOST model is used to calculate the transport of P from each sub-catchment based on the region the sub-catchment is located within and additional information for each particular sub-catchment: soil types, crop distribution, point sources, and mitigation measures. Transport of P is recalculated to eliminate the effect of existing buffer zones in the catchment, and this P-transport baseline is then imported into FyrisSKZ to use for calculating the effect of buffer zones in the sub-catchment. Where there is a potential for a buffer zone (the light-blue areas in Fig. 2), the P reduction of a buffer zone of a particular width (2, 6, 10, 15 and 20 m) is estimated for the FyrisSKZ tool by using P loss coefficients from NLeCCS/IcecreamDB (see Fig. 1) for each width and slope class and parameterized for one crop type with climate data from one region. For example, the reduction figures in Table 1 are the IcecreamDB estimated percentage reduction effect of P-transport on three slope types in the study area described below when there is a buffer zone along a watercourse.

**Table 1:** Estimated % P reduction effect of five buffer zone widths on three classes of slope

Buffer zone width	2 m	6 m	10 m	15 m	20 m
Sl 1 (0–1.99 %)	−13 %	−29 %	−42 %	−52 %	−60 %
Sl 2 (1.99–3.26 %)	−18 %	−36 %	−48 %	−58 %	−65 %
Sl 3 (>3.26 %)	−27 %	−46 %	−58 %	−66 %	−72 %

The economic information entered directly into the FyrisSKZ tool (Fig. 1) represents two types of costs: opportunity costs for converting productive agricultural land into a buffer zone, and establishment and maintenance costs for the area of a buffer zone. The value of land is assigned for each of the eight Swedish production regions based on leasing costs from agricultural land reported to Statistics Sweden. As around 40 % of Swedish agricultural land is leased, there is a great deal of data on leasing costs. Unfortunately there is also a large spread in prices reported. The FyrisSKZ tool uses the 90th percentile of the reported prices in order to capture the effect that the decision to establish a buffer zone competes with other land uses. For landowners to be willing to establish a buffer zone, they must be offered a price that is sufficient to compensate them for their loss in production. An average price would only be high enough to compensate 50 % of the landowners and lead to a lower landowner interest in establishing a buffer zone, an effect that would be even more pronounced the more productive the land is. Establishment costs for a buffer zone were estimated by the Swedish Board of Agriculture for sowing, seed, and labor to be €17 per year ha^−1^ based on a 5-year period. The FyrisSKZ tool uses the same annual cost for establishment as it is assumed that these costs do not vary significantly across the country. The costs for each of the eight Swedish production regions for estimating cost efficiency of buffer zones with the FyrisSKZ tool are reproduced in Table 2.

**Table 2:** Annualized leasing prices for agricultural land and establishment costs for buffer zones for the 8 production regions (PO8) in FyrisSKZ. Production regions are ordered from the highest to lowest leasing prices which in Sweden follow a general south to north gradient

	Leasing price (€ ha^−1^ yr^−1^)	Establishment cost (€ ha^−1^ yr^−1^)	Total annual cost (€ ha^−1^ yr^−1^)
Götalands södra slättbygder	702	17	719
Götalands mellanbygder	445	17	462
Götalands norra slättbygder	330	17	347
Svealands slättbygder	217	17	234
Götalands skogsbygder	217	17	234
Mellersta Sveriges skogdbygder	131	17	148
Nedre Norrland	97	17	114
Övre Norrland	78	17	95
Weighted average^a^	441	17	458

^a^Weighted by area of leased land in each region as a percentage of total leased land

### SKZ model application: The modeled cost efficiency of three buffer zone scenarios in the Svärta River Catchment

To illustrate how the FyrisSKZ tool can support policy analysis, three scenarios for allocations of riparian buffer zones in the Svärta River catchment were evaluated. The Svärta River catchment is located in central Sweden south of Stockholm and drains directly to the Baltic Sea. Of the total catchment land area of 345 km^2^ about 25 % is used for agriculture (9000 ha) with 7500 ha of this in crop production. There are two dominant soil types in the catchment: silty clay loam (80 %) and silty loam. The majority of the soil has a high soil P concentration. As described above, the FyrisCOST model uses a three-tier system for P classes, and of the 13 sub-catchments in the Svärta River catchment, there are 11 of these in the highest class, 1 in the middle, and 1 in the lowest class. Erosion sensitive agricultural land (0–50 m from watercourses) is also divided into three slope classes with a distribution in the catchment of 11 in the highest class (greatest slope) and 2 in the middle class.

The baseline scenario (Scenario 1) in the study uses the actual distribution of buffer zones which received subsidies from the Swedish RDP in 2008 and estimates the effect and costs of buffer zones used for calculation of the HELCOM PLC5 data (10 m wide) using output from the FyrisSKZ tool. The PLC5 data used a 10 m buffer zone width for all of the buffer zones because input data were only available for the total area of buffer zones subsidized through the RDP in each sub-catchment and not for specific widths (from 6 to 20 m) that were eligible under program guidelines. Scenario 2 allocates a 6-m-wide buffer zone on all the potential area in each sub-catchment. Potential area is based on the requirements for buffer zones along watercourses as defined in the Swedish RDP guidelines and estimated using the FyrisSKZ tool. Scenario 3 allocates areas of buffer zones (widths) to sub-catchments based on the lowest cost of reduction to achieve a total P reduction similar to the baseline scenario (Scenario 1).

## Results and discussion

Table 3 summarizes the results from the three scenarios. In the second column of Table 3, the cost per hectare of buffer zones in the Svärta River catchment is the same for each of the three scenarios (Svealands slättbygder in Table 2). This is because for each of the eight production regions of Sweden, there is only regional data available for leasing (land) costs. However, based on available bio-physical and agronomic inputs, the estimated total reduction differs under each scenario and leads to a spread in total costs and average costs for the three scenarios. Under the baseline scenario (Scenario 1), the 162 ha of buffer zones in the RDP resulted in a reduction of total P of just below 100 kg for the catchment. The average cost per unit of reduction for the baseline scenario is €390 kg P^−1^ with an average reduction of 0.60 kg P for every hectare in the program. The total P reduction is increased by 30 % in Scenario 2 compared to the baseline scenario, and the average reduction per hectare increases by 90 % while the total costs of the program fall by 32 %. The increased reduction and the constant cost per hectare of the land in buffer zones lead to a 45 % fall in the average cost per unit of P reduced: from €390 to €207 kg P^−1^. In the third scenario, the average cost per unit of reduction (€163 kg P^−1^) is the lowest of the three scenarios: 58 % lower than the baseline and 21 % lower than Scenario 2. In addition, as a result of having the lowest amount of land in buffer zones (71.5 ha), this scenario also has the lowest total program costs: 56 % lower than the baseline and 35 % lower than Scenario 2.

**Table 3:** Scenario allocation results

	Buffer zone area (ha)	Cost per ha (€)	Total cost (€)	Total P reduction (kg)	Average P reduction (kg ha^−1^)	Average cost (€ kg P^−1^)
Scenario 1	162	234	37 908	97.2	0.60	390
Scenario 2	110	234	25 740	124.5	1.13	207
Scenario 3	71.5	234	16 731	102	1.42	163

In Scenario 3, a program targeted at implementing 6 m buffer zones in all potential areas in six sub-catchments results in a similar reduction to the baseline scenario at a lower cost and at a lower cost than having 6 m zones in all the sub-catchments as under Scenario 2. In spite of the lowest average cost per unit of P reduction in Scenario 3, it is not clear that this is the most cost efficient of the three scenarios. Selecting the most cost efficient scenario depends on the goal set by program administrators. If the goal is to maximize total P reduction, then Scenario 2 would be the preferred scenario as this leads to a higher reduction compared to the other two scenarios. However, if the goal is to achieve the lowest cost per unit of reduction, then Scenario 3 is the preferred scenario. The targets for P reduction under the BSAP would tend to support the adoption of Scenario 2 as this provides the greatest total reduction.

Using a uniform payment to landowners, as assumed under all three scenarios, may make it difficult to achieve voluntary adoption of 6-m-wide zones everywhere in the catchment where there is a potential for a buffer zone. Voluntary programs require sufficient payment and sufficient information to achieve expected uptake levels. While the second of these two requirements would increase the transaction costs for a targeted program, reducing the total cost of the program (Scenarios 2 and 3 compared to the baseline) could make this possible. For example, under the RDP program in 2008, the total costs for buffer zones in the Svärta River catchment were around €38 000 while under Scenario 3, these were less than €17 000. The €21 000 difference in the two program costs could be used to promote adoption in the six targeted sub-catchments. This could perhaps be in the form of targeted media campaigns, visits to landowners by agricultural advisors or some other type of informational outreach. The problem of payment sufficient to induce participation is in part addressed by using a land cost based on reported leasing costs at the 90th percentile. While using this land cost may result in economic rents for some landowners with marginally productive land, it may be adequate for including the majority of productive land in a program. An alternative would be to adopt a policy which allowed for individually negotiated land payments, but this could also significantly raise transaction costs and lead to lower cost efficiency.

Finally, it is important to point out that the two alternative scenarios evaluated (Scenarios 2 and 3) are only a subset of the total number of possible scenarios. The types of programs evaluated could be extended to include other targets. For example, if the administrative target was to achieve the same P reduction as under the baseline scenario for the region covered by one of the Swedish Water Authorities, this would include a large number of catchment areas and may lead to some catchments with no reduction and other catchments with greater reductions than if the target was set for each individual catchment. There are also other scenarios that could be of interest based on other criteria. Regardless of the type of program evaluated, since the FyrisSKZ tool covers all the 12 864 sub-catchments in Sweden, it is possible to evaluate and compare any type of program at any scale above the level of a single sub-catchment.

Tools similar to FyrisSKZ could be developed for evaluation and design of other agricultural mitigation programs. Current plans include developing tools based on the same platform for evaluating two types of measures: catch crops and structural liming. The possibility of not only evaluating single programs of measures but also combinations of measures would make it possible for local authorities to design cost efficient action programs for meeting the targets of the WFD and the BSAP. If similar types of models and sufficient data were available, the FyrisSKZ type of tool could be developed in other countries.
